# Cellular Activity of *Salmonella* Typhimurium ArtAB Toxin and Its Receptor-Binding Subunit

**DOI:** 10.3390/toxins13090599

**Published:** 2021-08-27

**Authors:** Elise Overgaard, Brad Morris, Omid Mohammad Mousa, Emily Price, Adriana Rodriguez, Leyla Cufurovic, Richard S. Beard, Juliette K. Tinker

**Affiliations:** 1 Biomolecular Sciences Graduate Program, Boise State University, Boise, ID 83725, USA; eliseovergaard@boisestate.edu; 2 Department of Biology, Boise State University, Boise, ID 83725, USA; bradmorris@u.boisestate.edu (B.M.); omidmohammadmousa@u.boisestate.edu (O.M.M.); adrianarodriguez@u.boisestate.edu (A.R.); leylacufurovic@u.boisestate.edu (L.C.); 3 Idaho Veterans Research and Education Foundation, Infectious Diseases Section, Boise, ID 83702, USA; Emily.Price@va.gov; 4 Biomolecular Research Center, Boise State University, Boise, ID 83725, USA; richardbeard@boisestate.edu

**Keywords:** *Salmonella* Typhimurium, AB_5_ toxin, ArtAB

## Abstract

Salmonellosis is among the most reported foodborne illnesses in the United States. The *Salmonella* *enterica* Typhimurium DT104 phage type, which is associated with multidrug-resistant disease in humans and animals, possesses an ADP-ribosylating toxin called ArtAB. Full-length *art*AB has been found on a number of broad-host-range non-typhoidal *Salmonella* species and serovars. ArtAB is also homologous to many AB_5_ toxins from diverse Gram-negative pathogens, including cholera toxin (CT) and pertussis toxin (PT), and may be involved in *Salmonella* pathogenesis, however, in vitro cellular toxicity of ArtAB has not been characterized. *art*AB was cloned into *E. coli* and initially isolated using a histidine tag (ArtABHIS) and nickel chromatography. ArtABHIS was found to bind to African green monkey kidney epithelial (Vero) cells using confocal microscopy and to interact with glycans present on fetuin and monosialotetrahexosylganglioside (GM1) using ELISA. Untagged, or native, holotoxin (ArtAB), and the pentameric receptor-binding subunit (ArtB) were purified from *E. coli* using fetuin and d-galactose affinity chromatography. ArtAB and ArtB metabolic and cytotoxic activities were determined using Vero and Chinese hamster ovary (CHO) epithelial cells. Vero cells were more sensitive to ArtAB, however, incubation with both cell types revealed only partial cytotoxicity over 72 h, similar to that induced by CT. ArtAB induced a distinctive clustering phenotype on CHO cells over 72 h, similar to PT, and an elongated phenotype on Vero cells, similar to CT. The ArtB binding subunit alone also had a cytotoxic effect on CHO cells and induced morphological rounding. Results indicate that this toxin induces distinctive cellular outcomes. Continued biological characterization of ArtAB will advance efforts to prevent disease caused by non-typhoidal *Salmonella*.

## 1. Introduction

Salmonellosis, caused by the Gram-negative bacteria *Salmonella*, is one of the most common foodborne diseases in the world [1,2]. Non-typhoidal serovars of *Salmonella* (NTS) generally cause self-limiting gastroenteritis. However, they can cause systemic infections and are increasingly antibiotic resistant, resulting in an estimated 1.35 million illnesses and 420 deaths per year in the U.S. [3]. *Salmonella* has a complex taxonomy and is a diverse human and animal pathogen. *Salmonella enterica*, one of two overall species, has six subspecies. One subspecies, *enterica* (*Salmonella enterica*, subsp. *enterica*), contains over 2500 serovars. The NTS within this subspecies are pathogenic to a broad range of vertebrate species, including humans and human food sources such as cattle, swine, and poultry [4,5]. This is in contrast to the narrow host range of the human typhoidal serovars *S*. Typhi and *S*. Paratyphi, which cause severe invasive disease with a high mortality rate but with lower numbers of cases globally.

The virulence factors contributing to *Salmonella enterica* pathogenicity are numerous and include well-characterized secretion systems, adhesins, and capsular polysaccharides [6]. In the mid-1990s, the NTS phage-type *S*. Typhimurium DT104 became the predominant strain infecting livestock and was commonly associated with human hospitalization [7]. DT104 is highly antibiotic resistant and virulent, however, studies have failed to fully identify the mechanism of increased virulence [8,9]. In 2005, DT104 was found to contain an enterotoxin, with homology to AB_5_-type toxins, called *art*AB [10]. AB_5_-type toxins are potent virulence factors produced by many pathogenic Gram-negative bacteria. They are composed of a pentameric B subunit (B_5_), which binds to host cell-surface receptors, and an A subunit, which becomes catalytically active inside the host cell. AB_5_-type toxins contribute to the development of infectious diseases associated with altered ion flow across membranes and disruption of epithelial barrier function [11,12]. *art*AB has since been identified on a number of additional *S. enterica* serotypes, and on *S. bongori,* and has been determined to be a secreted ADP-ribosylating toxin with homology to the AB_5_-type toxins cholera toxin (CT) and pertussis toxin (PT) [13,14,15].

CT and PT are two of the most well-characterized bacterial toxins. CT, produced by *Vibrio cholerae*, is folded into a holotoxin in the bacterial periplasmic space prior to secretion and interaction with the host cell. CT becomes activated when the active subunit (CTA) is proteolytically cleaved, or nicked, by a host serine protease [16]. After secretion, the binding subunit (CTB) binds to monosialotetrahexosylganglioside (GM1), which is expressed on the surface of intestinal epithelial cells. This interaction triggers retrograde endocytosis of the CT holotoxin to the endoplasmic reticulum (ER). In the ER, CTA becomes separated from CTB and moves into the host cell cytoplasm where it binds to, and constitutively activates, the Gα_s_ protein through ADP ribosylation. Gα_s_ activates adenylate cyclase (AC), causing an increase in cyclic adenosine monophosphate (cAMP), which leads to dysregulation of cellular ion channels and, ultimately, secretory diarrhea. PT, produced by *Bordetella pertussis*, is a homologous AB_5_ toxin with mechanistic similarities. However, the B subunit is structurally distinct and binds to a broader range of sialylated and non-sialylated *N*-linked glycans [17]. PT’s active subunit (PTA) has a distinct ADP-ribosylation target; it inactivates Gα_i_. Despite having different ADP-ribosylation targets, the outcome is similar; AC is activated, and cellular cAMP is increased. CT and PT are also both components of licensed safe and effective vaccines [18,19]. The ability to chemically or genetically detoxify CT, PT, and other AB_5_ toxins while retaining antigenicity and immunomodulatory characteristics has supported their use as vaccine adjuvants and molecular tools for cellular delivery [20,21,22,23]. Thus, in addition to improving the understanding of *Salmonella* pathogenesis, the study of the cellular activities of ArtAB and ArtB is relevant to vaccine design and development.

While the ADP-ribosylation activity and in vivo lethality of ArtAB have been reported, the cellular activity, including the cytotoxicity and morphologic activities, of ArtAB and its receptor-binding subunit, ArtB, have not been previously described [14,15]. In addition, despite numerous studies on various AB_5_-type toxins, few studies have directly compared the cellular effects of these toxins. The goals of this study were to develop a rapid method of purification of ArtAB and ArtB from *E. coli* and to use the purified proteins to identify a reproducible and consistent cellular phenotype in vitro. Results indicate that ArtAB induces a slow cytotoxic response and characteristic changes in cellular morphology, similar to the responses induced by CT and PT. ArtB alone may also be cytotoxic, and it induces a distinct morphologic response. These studies will promote further structural and functional characterization of ArtAB and improve our understanding of its role in *Salmonella* pathogenesis.

## 2. Results

### 2.1. Phylogenetic Analysis of AB_5_ Toxin Subunits

**Figure 1:**
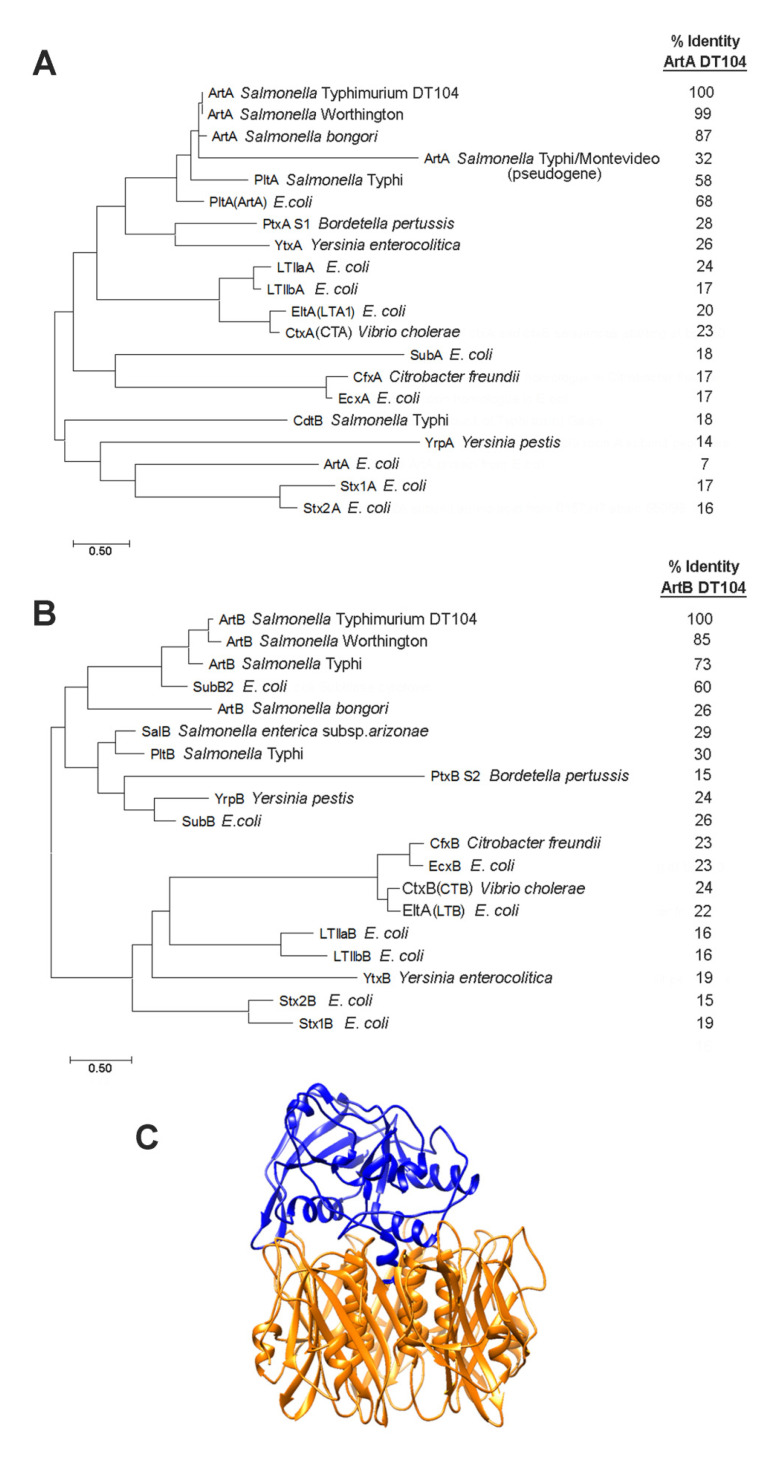
Phylogeny and predicted tertiary structure of ArtAB and closely related AB_5_ toxins. (**A**) phylogenetic analysis of enzymatically active (A) subunits of AB_5_ toxins, and (**B**) binding (B) subunits of AB_5_ toxins, as constructed with MEGA7 software using a maximum-likelihood method based on the JTT matrix-based model [24]. (**C**) I-TASSER *Salmonella* DT104 ArtAB tertiary structure prediction based on *E. coli* SubB and *S*. Typhi PltA subunit crystal structures showing the pentameric B subunit (gold) and the active A subunit (blue) [32].

Phylogenetic trees were constructed with MEGA using the maximum-likelihood method to align the amino acid sequences of *S*. Typhimurium DT104 ArtA and ArtB to those of AB_5_ toxin subunits from other Gram-negative bacteria [24]. As shown in Figure 1A, the enzymatically active ArtA subunit is most closely related to ArtA from *S*. Worthington and *S. bongori*, as described [14]. *S*. Typhimurium DT104 ArtA is also related to the active subunit of an AB_5_ toxin from *E. coli* (68% identity), which has been named previously as both pertussis-like toxin (*Ec*PltA) and ArtA [25,26]. *S*. Typhimurium DT104 ArtA also has homology (58% identity) to *S*. Typhi PltA, an active subunit of the *S*. Typhi pertussis-like toxin (Plt). Plt is also commonly referred to as typhoid toxin (TT). Plt/TT is a unique A_2_B_5_ toxin that utilizes two active subunits: PltA and CdtB. CdtB has nuclease activity and low homology (18% identity) to *S*. Typhimurium DT104 ArtA [27]. ArtA also shows significant relatedness to a pseudogene found in both *S*. Typhi and *S*. Montevideo (90% identity over a 99 amino acid central region), that is unlikely to be expressed [28]. Lastly, ArtA is distantly related to the active subunits of PT (PTA S1; 28% identity), an uncharacterized AB_5_ toxin from *Yersinia* (YtxA; 26% identity), *E. coli* type IIa heat-labile enterotoxin (LTIIaA; 24% identity), and CT (CTA; 23% identity).

Figure 1B shows the phylogenetic relationships of *S*. Typhimurium DT104 ArtB, which is most closely related to the B subunits from *S*. Worthington and *S*. Typhi (85 and 73% identity, respectfully). An uncharacterized subtilase cytotoxin-like binding subunit (SubB2) from *E. coli* shows 60% identity to *S*. Typhimurium DT104 ArtB, while the more well characterized *E. coli* subtilase cytotoxin B (SubB), which has been shown to promote the binding to sialylated glycans, has only limited homology (26% identity) [29]. *S*. Typhimurium DT104 ArtB has low homology to the binding subunit of Plt/TT (PltB; 30% identity), supporting previous glycan array studies that revealed ArtB’s specificity for sialylated glycans that are distinct from those that bind with PltB [30,31]. *S*. Typhimurium ArtB has more limited homology to the well-characterized binding subunit of CT (CTB; 24% identity), as well as to the likely AB_5_ toxins from *S. arizonae* (SalB; 29% identity), *S. bongori* (ArtB; 26% identity), and *Yersinia pestis* (YrpB; 24% identity). PT has four distinct binding subunits, and ArtB has some limited homology to the second (PtxB S2; 15% identity). A ribbon diagram of the predicted structure of ArtAB was constructed using I-TASSER and is shown in Figure 1C [32,33].

The *art*AB-like operons from a non-DT104 *S*. Typhimurium isolate, as well as *S*. Typhi, *S*. Paratyphi, and *S*. Choleraesuis were analyzed by PCR and compared to four human clinical *S*. Typhimurium DT104 isolates using flanking primers of the *art*AB region (Supplementary Figure S1A). The smaller amplicons of 957 and 954 bp, respectively, support the presence of the homologous *art*B gene and a truncated pseudogene which contains a deletion within *art*A (Supplementary Figure S1B).

### 2.2. Purification and Binding of ArtABHIS

*S*. Typhimurium DT104 *art*AB was cloned into the pBAD18 expression vector with a hexa-histidine tag (6XHIS) introduced at the C-terminus of the B subunit to construct pBM003 (Supplementary Figure S2A). Holotoxin was purified using cobalt affinity chromatography. Purified toxin with the expected molecular weights of processed (signal sequence removed) ArtA (~25.6 kDa) and ArtB-HIS (~14.2 kDa) were confirmed using SDS-PAGE, western blotting (Supplementary Figure S2B), and protein sequencing by mass spectrometry (Supplementary Table S2). A consistently co-purified, but unexpected, second small peptide (~13 kDa) was also confirmed to be ArtB by protein sequencing (Supplementary Figure S2C, Supplementary Table S2). This peptide was not HIS-tagged, as determined by anti-6XHIS western blot (Supplementary Figure S2B).

**Figure 2:**
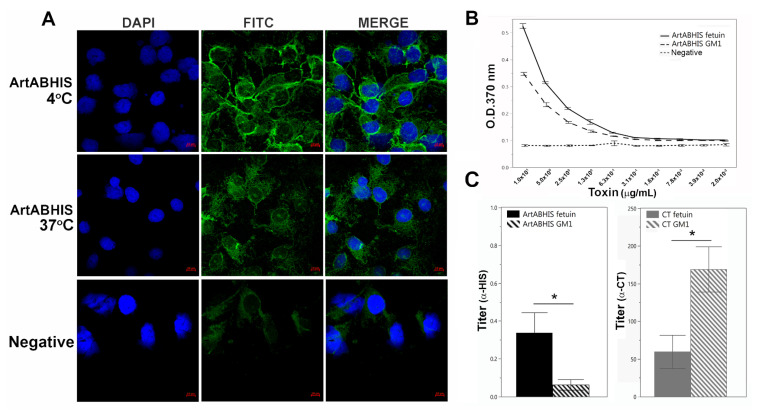
Characterization of ArtABHIS purified from *E. coli*. (**A**) binding (at 4 °C) and uptake (at 37 °C) of 50 μg/mL ArtABHIS after 1 h on Vero cells using anti-6XHIS (α-HIS) and FITC-conjugated secondary antibodies (negative = no toxin control, scale bar = 10 μm), (**B**) representative ELISA dilution series of ArtABHIS on ganglioside GM1 and fetuin (negative = no toxin control), and (**C**) average titers of fetuin and GM1 ELISAs of ArtABHIS using α-HIS and CT using anti-CT (α-CT) antibody. Results show the mean +/− SE (*n* = 4). A two-tailed, unpaired *t*-test was performed for comparison within each toxin group (ArtABHIS fetuin to GM1 * *p* = 0.0487; CT fetuin to GM1 * *p* = 0.0262).

We assessed the binding of ArtABHIS to epithelial cells in vitro using fluorescence microscopy. After 1 h at 4 °C to block endocytosis, ArtABHIS was found to be surface-associated on African green monkey kidney epithelial (Vero) cells. After 1 h at 37 °C, the toxin was no longer visible on the cell surface, but microscopy revealed potential punctate staining within the cell that was above background levels (Figure 2A).

To begin to assess the binding specificity of ArtABHIS, we performed ELISA-based binding assays with purified ArtABHIS. Since CT is known to have a strong binding affinity for GM1 and some affinity for the sialylated glycans found on the blood protein fetuin, we performed a comparative assay with these two receptor molecules using anti-6XHIS (for ArtAB) or anti-CT (for CT) antibodies [34,35]. ArtABHIS bound to fetuin with consistently higher titers than to GM1 (Figure 2B,C), while CT bound to GM1 with higher average titers (Figure 2C).

### 2.3. Cloning and Glycan Affinity Purification of ArtAB and ArtB

**Figure 3:**
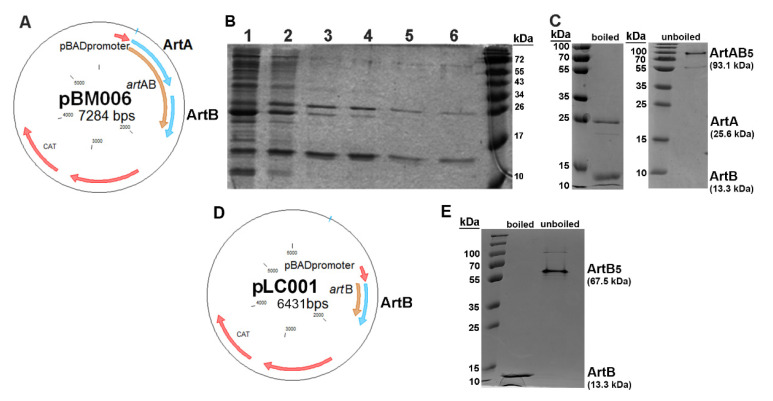
Purification of ArtAB and ArtB from *E. coli*. (**A**) plasmid pBM006 for expression of ArtAB, (**B**) SDS-PAGE of ArtAB purified with fetuin, showing increasing salt fractions (*1*: flow through, *2*: 0.1 M NaCl, *3*: 0.1 M MgCl_2_, *4*: 0.5M NaCl, *5*: 0.5M MgCl_2_, *6*: 1 M NaCl). (**C**) SDS-PAGE of the ArtAB elution purified with d-galactose, and boiled/unboiled samples, (**D**) plasmid pLC001 for expression of ArtB and (**E**) SDS-PAGE of the ArtB elution purified on d-galactose, boiled and unboiled samples.

To eliminate potential interference of the affinity tag with toxin assembly and receptor binding, native (untagged) ArtAB was purified using glycan affinity chromatography. This approach was based on methods of purification of other AB_5_ toxins and the observed binding of ArtABHIS to fetuin and GM1. The plasmid pBM006 (Figure 3A) was constructed to express untagged *S*. Typhimurium DT104 ArtAB, and the toxin was purified using fetuin affinity chromatography with increasing salt concentrations based on reported protocols for PT [36]. Purified holotoxin with the expected subunit molecular weights of ArtA (≅ 25.6 kDa) and ArtB (≅ 13.3 kDa) was confirmed using SDS-PAGE (Figure 3B) and mass spectrometry (Supplementary Table S2).

As determined from ArtABHIS binding assays, ArtAB also binds to ganglioside GM1. GM1 contains d-galactose moieties, thus ArtAB holotoxin was also purified using d-galactose affinity chromatography. Purified ArtAB was confirmed with SDS-PAGE (Figure 3C), and unboiled samples supported an AB_5_ compostion of approximately 93 kDa. Despite evidence that ArtAB binds less well to GM1, native ArtAB isolated using immobilized d-galactose was of markedly higher purity than native ArtAB isolated on fetuin, and had higher purification efficiency, with close to 6 mg of protein purified from one liter of culture (versus 2–4 mg/liter using fetuin). SDS-PAGE of fetuin-purified ArtAB, and sometimes d-galactose-purified ArtAB, revealed a third peptide that was similar in molecular weight to ArtA (Figure 3B, lanes 3–4). All peptide bands, including the two ArtA bands that purified near 25 kDa, were sequenced by LC–MS mass spectrometry to confirm identity (Supplementary Figure S2D, Supplementary Table S2). The larger (~26 kDa) and smaller (~23 kDa) peptides were both confirmed to be ArtA, indicating that the A subunit may also be proteolytically cleaved, or nicked, similar to that observed for PT [37].

Purification of the ArtB binding subunit alone was also completed using d-galactose affinity chromatography. The plasmid pLC001 (Figure 3D), was constructed to express *S*. Typhimurium DT104 ArtB alone from *E. coli*. d-galactose-isolated ArtB was observed using SDS-PAGE and unboiled samples were consistent with a pentameric structure of approximately 67 kDa (Figure 3E). Purified ArtB was also confirmed by mass spectrometry (Supplementary Table S2).

### 2.4. Cellular Activity of ArtAB

**Figure 4:**
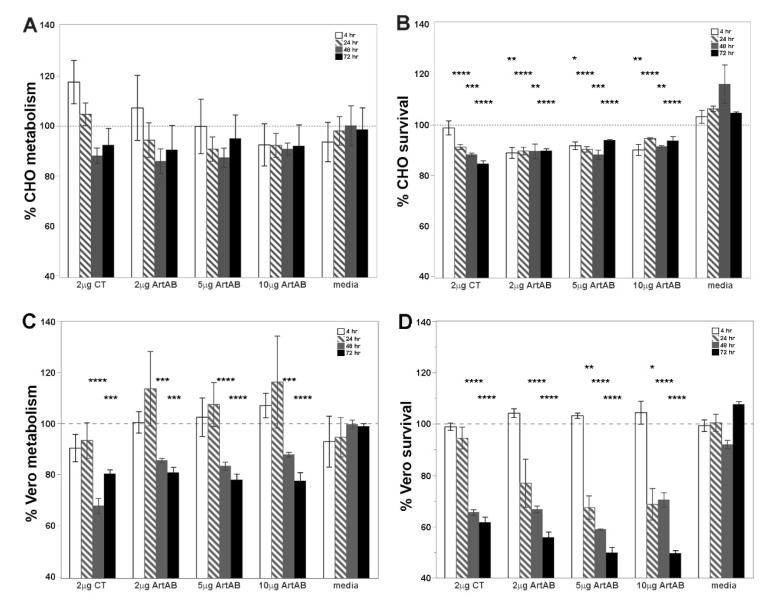
Metabolic and cytotoxic activity of ArtAB and CT on epithelial cells in vitro. (**A**) Resazurin metabolic assays and (**B**) crystal violet cytotoxicity assays on CHO cells, and (**C**) resazurin and (**D**) crystal violet assays on Vero cells. Purified ArtAB was incubated with cells at 2, 5, or 10 μg per 200 μL well for 4, 24, 48, or 72 h at 37 °C. Results are reported as the percent of vehicle control activity, and toxin groups at each time point are compared to media alone (also reported as the percent of vehicle control activity) using a one-way analysis of variance (ANOVA) and Tukey’s HSD. Stars above the bar indicate a significant difference from media alone at that time point (* *p* ≤ 0.05, ** *p* ≤ 0.01, *** *p* ≤ 0.001, **** *p* ≤ 0.0001).

The cellular activity of purified ArtAB was determined using two epithelial cell lines. Vero and Chinese hamster ovary (CHO) epithelial cells have long been used to characterize the effects of AB_5_-type toxins such as CT and PT [38,39]. The cellular activity of three concentrations of purified ArtAB on Vero and CHO cells over 72 h of incubation was assessed (Figure 4). Metabolic activity was determined using resazurin dye (alamarBlue^TM^), and cytotoxicity was determined using crystal violet, as described [40]. The effect of each concentration of toxin is expressed as a percent of the effect of vehicle alone.

On CHO cells, metabolic assays at early time points indicated that ArtAB had no effect, or potentially increased the metabolic activity (Figure 4A). At later time points, metabolic activity dropped to an average of 90% of vehicle control (lowest activity was 85.8% of control with 2 μg at 48 h). Cytotoxicity assays on CHO cells treated with holotoxins were consistent with these results, with significant differences between holotoxin-treated and vehicle-treated cells at all time points (Figure 4B). Vero cells revealed a higher sensitivity to ArtAB with metabolic activity dropping to an average of 80% of vehicle control (lowest activity was 77% of control with 10 μg at 72 h), and significant differences between holotoxin-treated and untreated cells at 48 and 72 h (Figure 4C). Vero cell cytotoxicity reached greater than 50% at later time points, with significant differences between holotoxin-treated and untreated cells at 24, 48 and 72 h (Figure 4D). There was no significant dose-dependent effect of ArtAB for either cell type in the range used (2 μg to 10 μg of toxin) in these assays. The metabolic and cytotoxic activity of 2 μg of ArtAB was simlar to that of 2 μg of CT on both cell types (Figure 4A–D). The cytotoxicity of ArtAB on Vero cells was significantly greater than that of 2 μg of CT at 5 μg (*p* = 0.0030) and 10 μg (*p* = 0.0025). PT was consistently more active on CHO cells than ArtAB, reaching below 80% of untreated cells for metabolic and cytotoxic activity with 2 μg of protein (Supplementary Figure S3).

**Figure 5:**
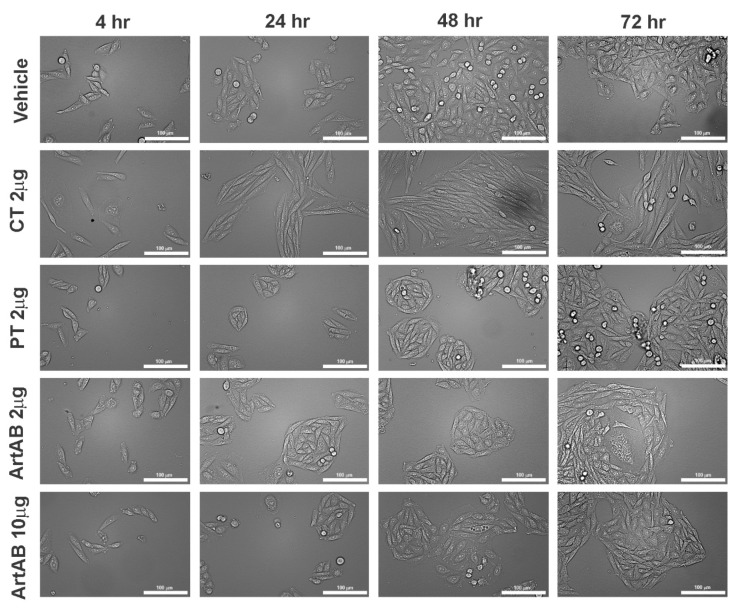
Cellular aggregate and morphology of CHO cells incubated with AB_5_ toxins. CHO cells were incubated with ArtAB (2 μg and 10 μg), CT (2 μg) and PT (2 μg) at 4, 24, 48 and 72 h. All images collected using brightfield microscopy (20×, scale bar = 100 μm).

Cellular morphological changes in response to treatment with ArtAB were examined using light microscopy. Treatment with ArtAB caused CHO cells to exhibit some elongation and a definitive clustering morphological phenotype compared to vehicle-treated cells beginning at 24 h. This response was similar to that induced by 2 μg of PT (Figure 5). By 72 h, CHO cell clusters were larger and less distinct for both toxins. In contrast, CT induced a distinctive elongated phenotype on CHO cells that was observable through 72 h. Cell length was quantified from representative images and, while significant CHO cell clustering effects by ArtAB were not identified using this method, CT elongation was highly consistent and easily quantified (Supplementary Figure S4A).

**Figure 6:**
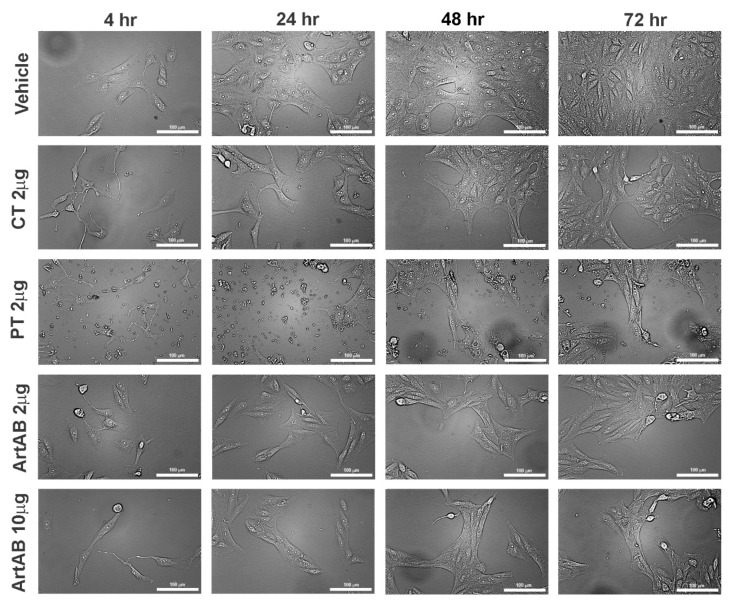
Cellular aggregate and morphology of Vero cells incubated with AB_5_ toxins. Vero cells incubated with ArtAB (2 μg and 10 μg), CT (2 μg) and PT (2 μg) at 4, 24, 48 and 72 h. All images collected using brightfield microscopy (20×, scale bar = 100 μm).

On Vero cells, all ArtAB treatments triggered varying levels of destruction of the cells with a less distinct phenotype. However, Vero cells treated with ArtAB demonstrated elongation and potential dendrite formation at early time points and throughout the 72 h incubation (Figure 6). This result was similar to that observed with CT at 2 μg. In contrast, PT treatment of Vero cells at 2 μg caused a very high amount of cellular destruction, even at early time points, with some cell populations able to recover by 72 h. Quantification of images verified a significant elongation effect of ArtAB treatment on these cells (Supplementary Figure S4B).

### 2.5. Cellular Activity of ArtB

**Figure 7:**
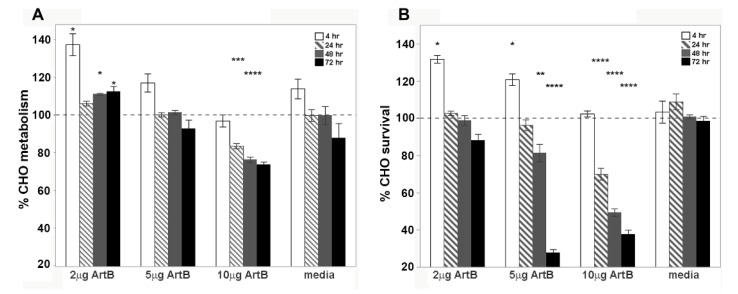
Metabolic and cytotoxic activity of the ArtB subunit on epithelial cells in vitro. (**A**) Resazurin metabolic assays and (**B**) crystal violet cytotoxicity assays on CHO cells. Purified ArtB was incubated with cells at 2, 5, or 10 μg per 200 μL well for 4, 24, 48, or 72 h at 37 °C. Results are reported as the percent of vehicle control activity, and toxin groups are compared to media alone at each time point (also reported as the percent of vehicle control activity) using a one-way analysis of variance (ANOVA) and Tukey’s HSD. Stars above the bar indicate a significant difference from media alone at that time point (* *p* ≤ 0.05, ** *p* ≤ 0.01, *** *p* ≤ 0.001, **** *p* ≤0.0001).

**Figure 8:**
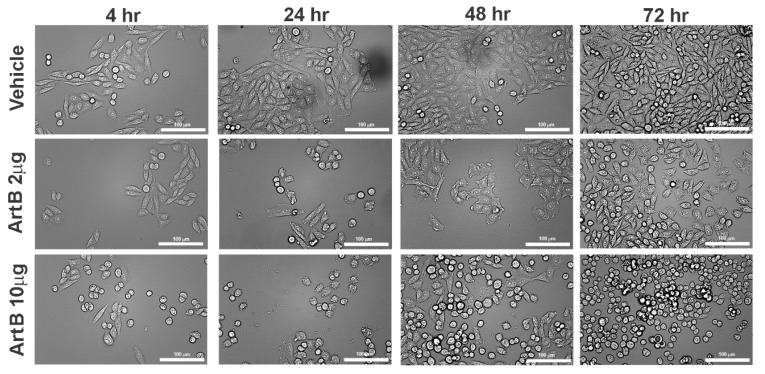
Cellular aggregate and morphology of CHO cells incubated with the ArtB subunit. CHO cells incubated with ArtB (2 μg and 10 μg) at 4, 24, 48 and 72 h. All images collected using brightfield microscopy (20×, scale bar = 100 μm).

The metabolic and cytotoxic activity of ArtB was determined using the same epithelial cell lines. Similar to holotoxin, ArtB induced an increase in metabolic activity at early time points that was significant over media alone for the lower 2 μg concentration (Figure 7A). At higher concentrations and later time points, ArtB significantly slowed metabolic activity and induced cell death, with percent survival below 40% at higher concentrations after 72 h (Figure 7B). ArtB also induced a significant concentration-dependent effect on both metabolism and cell survival at all time points. Visual assessment of cells by microscopy indicated that ArtB alone induces morphologic changes on CHO cells that are distinct from those induced by holotoxin. Treatment with ArtB may have prevented adherent CHO cells from forming paracellular junctions, resulting in the consistent rounding of adherent cells (Figure 8). The quantification of cell length also supported the consistent rounding of CHO cells as a result of incubation with ArtB (Supplementary Figure S4C). Incubation of Vero cells with ArtB alone did not result in significant metabolic, cytotoxic, or morphologic cellular changes that were distinct from vehicle-treated cells alone (data not shown).

## 3. Discussion

We report the glycan affinity purification and cellular activity of *S*. Typhimurium ArtAB toxin as well as that of the ArtB binding subunit alone. The method of using d-galactose affinity for purification was found to be rapid and efficient and was used to purify native (untagged) ArtAB and ArtB to high concentrations. The cellular metabolic, cytotoxic, and morphologic effects were identified on two epithelial cell lines that have been commonly used to characterize bacterial toxins.

Native toxin purified from *E. coli* is consistent with the expected size and sequence of toxin purified directly from *S. enterica* Typhimurium DT104, enabling large scale protein purification to be performed in lower biohazard laboratory conditions. Unidentified differences in purification conditions resulted in the occasional production of a lower-molecular-weight peptide that was confirmed by LC–MS to also be ArtA (Supplementary Figure S2D), providing evidence that ArtA contains a proteolytic cleavage site. An A1 subunit of 22–23 kDa indicates that ArtA may contain a proteolytic processing site, similar to that reported for PT and CT [41,42]. The cleavage of CT is required for maximal activation and cellular activity. The enzyme for CT cleavage is a host-derived serine protease that generates a 22 kDa A_1_ subunit which remains connected to the A_2_ subunit via a disulfide bond [16]. Similar to CT, processing of PT is predicted to occur within a protease-sensitive loop of the S1 subunit near the C-terminus. The PT S1 subunit is thought to remain connected to the holotoxin through a disulfide bond; located between residues C41 and C201 [43]. In vitro, PT S1 cleavage results in a 22 kDa S1 subunit (from the 26 kDa full-length S1) and enhances ADP ribosylation [41]. While reduction of the disulfide bond and ATP-binding are both required for PT activity, proteolytic processing has not been found to be essential for cellular activity [44,45]. Our results indicate that the A subunit of ArtAB may be cleaved. However, current efforts have not identified a specific cleaving enzyme or determined if cleavage is required for activation. ArtA does contain cysteine residues (C38 and C198) in primary structure locations similar to those of PT, which may hold the cleaved strands of the A subunit together into the holotoxin structure.

Purification of hexa-histidine-tagged ArtAB resulted in the consistent production of two lower-molecular-weight bands under 15 kDa (Supplementary Figure S2C). Both peptides were confirmed by LC–MS to be ArtB, and the smaller band was untagged as determined by Western blot. We hypothesize that the location of the HIS-tag results in steric hindrance and may prevent pentamer formation with fully tagged monomers. While this toxin could be purified to high concentration, the location of the tag may reduce normal receptor binding and/or prevent glycan binding at a second site, which has been determined to be located on the outside of the pentamer [30]. Cell trafficking and receptor-binding assays reported here with the ArtABHIS protein are preliminary, and there is a possibility of non-specific interactions using α-HIS. Thusfuture studies will focus on the use of native toxin for these assays.

Cellular assays indicated that purified ArtAB induced a slow and limited cytotoxicity that is similar to that of CT. These studies revealed an approximate 15% (CHO) to 50% (Vero) decline in cell viability compared to vehicle-treated cells over 72 h. In dividing cells, alamarBlue™, or resazurin dye, is reduced to resorufin by aerobic respiration, so this assay is an indicator of metabolic activity rather than a comparison of live-versus-dead cells. Resazurin assays confirmed the decline in cell viability at later time points. Notably, we did not observe concentration-dependent cellular activity using a limited range of concentrations (2, 5, or 10 μg per 200 μL treatment). In addition, by 72 h, some cells in all cultures were able to recover from, or were initially resistant to, toxin treatment such that a detectable recovery of individual cells, as well as the population as a whole, could be observed. This is consistent with a bi-modal response to toxins that are able to bind to more than one cellular receptor, as has been described [46,47]. Binding to a sub-optimal host glycan may promote a lower concentration of toxin reaching the host cytosol and thus a population of cells that are not fully intoxicated. This effect may also explain why concentration-dependent responses cannot be detected unless concentrations cover a larger range. Future studies can assess a broader range and use assays to detect individual cell responses within a population.

The cellular aggregation and morphological changes observed in this study are consistent with a previous assay of the *Salmonella* supernatant activity on CHO cells and the identification of ADP-ribosylation activity [15]. These studies indicate that the observed cellular changes are induced by active toxin-stimulated increases in cAMP and that they are dependent upon specific active subunit–receptor interactions [48,49]. CT is well known to induce elongation in CHO cells and rounding in Y1 adrenal gland cells and has been reported to cause increased cellular adherence [50,51,52]. It has also been reported that adhesion molecules such as Thrombospondin-1 and Integrin-β1 are upregulated when monocytes are treated with CT [53]. In contrast, when treated with PT, CHO cells, which normally grow in uniform monolayers, exhibit a distinct clustering phenotype [54]. This is similar to our observations with ArtAB and supports the hypothesis that this toxin also induces a receptor-specific cellular activity that involves cytoskeletal rearrangement and changes in expression of adhesion molecules [55]. Two novel pertussis-toxin-like toxins from *E. coli* have been reported to induce both elongation and clustering of CHO cells [56]. These studies futher utilitize CHO morphology as a sensitive bioassay to determine toxin titer and identify potential receptors. The cellular phenotypes identified for ArtAB could similarly be used to assay toxin concentrations and identify receptors using mutant cell lines.

Somewhat surprisingly, the ArtB subunit alone also had cytotoxic activity on CHO cells, killing up to 60% of cells after 72 h at higher concentrations, as determined by crystal violet assay. The morphology of CHO cells was also affected by ArtB. We observed a distinct rounding of individual adherent cells, possibly indicating that ArtB is preventing the formation of paracellular junctions. These results indicate that receptor-binding and/or internalization of the B subunit alone induces significant cellular activity and may be relevant in bacterial pathogenicity. This has not been described for CTB but has been described for Shiga toxin B subunit [57]. *art*B is also present on typhoidal *Salmonella* serovars and is associated with an *art*A pseudogene that is likely not expressed. Recently *S*. Typhi *art*B has been found to be present on many additional *Salmonella* serovars and may provide an alternative binding subunit for Typhi Toxin (TT) [28]. *art*B of *S*. Typhi was also determined to be expressed, especially under conditions that may occur inside of host cells. While ArtB of *S*. Typhi has only 74% identity to ArtB of *S*. Typhimurium DT104 (Figure 1), these studies support the important role that the binding subunit alone may play in *Salmonella* pathogenesis. The crystal structure and glycan binding array of *S*. Typhimurium ArtB has been determined using a purified 6XHIS subunit from *E. coli* [30]. These studies indicated that ArtB likely binds to terminal Neu5Ac and Neu5Gc sialylated glycans, both of which can be found on bovine fetuin. GM1 is a monosialylated glycosphingolipid with an internal Neu5Ac, and a terminal d-galactose. d-galactose agarose affinity purification has long been established as a rapid and efficient method of purifying CT [58]. The ability of ArtAB to be efficiently purified using d-galactose indicates that this toxin also binds well to this terminal glycan and likely has a broad receptor specificity, similar to that of PT [17]. The binding to Neu5Gc also supports the importance of this toxin in animal disease, as humans do not synthesize this glycan. In these studies, we identified the binding and internalization of ArtABHIS into Vero cells, and activity on Vero and CHO cells. Future studies will assess the activity of ArtAB on additional cell types, as well as the binding to other glycoproteins/glycolipids, to help narrow potential cellular receptors, define target host cells and explore the utility of this toxin or receptor as a potential animal or human vaccine.

Both cell types incubated with ArtAB, and CHO cells incubated with ArtB, showed a potential increase in metabolic activity at early time points. This response was significant for ArtB on CHO cells and corresponded to an increase in cell number. The metabolic increase is consistent with the use of CT as an adjuvant that enhances the activation and proliferation of immune cells, as well as other cell types, at low concentrations [59,60,61]. This burst may also reflect the initiation of programmed cell death and the production of extracellular membrane vesicles, or exosomes, in response to intoxication. CT has been found to induce apoptosis in different cell types [62,63]. Vero cells in these studies were more sensitive to toxin and also produced a large number of exosomes in response to both CT and ArtAB. Exosomes can be induced as a survival mechanism to rapidly rid the cell of toxin or B subunit, as has been shown for *S. aureus* α-toxin [64]. We hypothesize that the mechanisms behind cell death and cellular defenses to ArtAB are similar to that of CT, and this toxin may induce apoptosis in some cell types. These activities will continue to be explored.

## 4. Conclusions

We report a method of rapid native ArtAB and ArtB purification based on glycan affinity and identify toxin binding and cellular activity in vitro. The availability of ArtAB and ArtB in native form will promote a varied set of downstream applications for the continued biological characterization of this toxin. Cellular assays can be used to further assess the expression of ArtAB from pathogenic *Salmonella* and the extent of ArtAB’s contribution to virulence and clinical outcomes in a broad host range. The structure and functionality of ArtAB can also be further defined, which will advance our understanding of bacterial AB_5_ toxins, as well as support efforts to prevent or treat salmonellosis in humans and animals. The identification of methods to reduce or eliminate *Salmonella* in agriculturally important animals is a priority, and will help to reduce animal mortality, increase production, and prevent transmission to humans.

## 5. Materials and Methods

### 5.1. Bacterial Strains and Growth Conditions

*Salmonellaenterica* Typhimurium phage-type DT104, isolates SC09039, SC09068, SC09073, SC09074, are human gastroenteritis clinical isolates obtained from the Idaho Bureau of Laboratories (Boise, ID) and used for genomic DNA preparation, *art*AB PCR, and sequencing. The isolate SC09039 was used for *art*AB and *art*B cloning and purification. Human clinical *S*. Typhi, *S*. Paratyphi A and *S*. Choleraesuis genomic DNA was supplied by the Idaho Bureau of Laboratories for PCR. *E. coli* TE1 [65] was used for cloning, and *E. coli* ClearColi^®^ BL21(DE3) (Lucigen, Madison, WI) was used for protein expression and toxin isolation. For genomic DNA preparation, bacterial cells were grown overnight in Luria Broth (LB) at 37 °C. For plasmid preparation, cells were grown in LB plus 25 μg/mL chloramphenicol (CM) and 0.02% glucose overnight at 37 °C.

### 5.2. Phylogenetic Analysis and Predicted Tertiary Structure of AB_5_ Toxin Subunits

Sequence alignments and evolutionary analysis were completed using MEGA 7 [24]. A rooted dendrogram of exhaustive pairwise AB_5_ toxin alignments of 20 sequences (A subunits) or 19 sequences (B subunits) was constructed using the maximum-likelihood method based on the JTT matrix-based model [66]. Protein sequences were retrieved from the National Center for Biotechnology Information NCBI. Available online: https://www.ncbi.nlm.nih.gov/ (accessed online on 22 May 2020) and accession numbers are provided in Supplementary Table S1. The I-TASSER server was used to visualize predicted tertiary structures of ArtA and ArtB using the model templates: *E. coli* SubB [29] for ArtB and *E. coli* PltA [25] for ArtA [32,33].

### 5.3. Construction of ArtABHIS, ArtAB, and ArtB Expression Plasmids

The *art*AB gene was amplified from *S*. Typhimurium DT104 SC09039 by polymerase chain reaction (PCR) from isolated genomic DNA using the 070pr forward primer (GATCCTCGCTAGCGTTT CTGTAGGAGGGTGTATG) and the 071pr reverse primer (GTACCAGAAGCTTTTAGTGATGGTGATGGT GATGGTTTGGCAACGTAGGTCCC), that introduces a 6XHIS. The amplified product was cloned into the pBAD18CM vector (ATCC, Manassas, VA) and transformed into *E. coli* TE1 for confirmation prior to introduction into the endotoxin-free *E. coli* strain ClearColi^®^ (Lucigen, Madison, WI) for protein production. The resulting plasmid (Supplementary Figure S2A) was designated pBM003. For pBM006 construction (Figure 3A), the *art*AB gene was amplified from DT104 SC09039 using the forward primer 070pr and the reverse primer 099pr (GCGCCAGAAGCTTGAAATATTTAGTTTGGCAAC GTAG). The amplified product was similarly cloned into pBAD18CM and transformed into *E. coli* TE1 and ClearColi^®^. To clone *art*B alone into pLC001 (Figure 3D), the forward primer, 198pr (GCCTAGGGCTAGCGGTAAATATTTTAGGAGTGG), which contains a modified ribosome-binding site, and the reverse primer, 099pr, were used to amplify *art*B from *S*. Typhimurium SC09039, and the resulting product was cloned into pBAD18CM and purified from ClearColi^®^. The sequences of pBM003, pBM006, and pLC001 were confirmed by sequencing through forward and reverse junctions (Idaho State University Molecular Research Core Facility, Pocatello, ID).

### 5.4. Expression and Purification of ArtABHIS, ArtAB, and ArtB from E. coli

ClearColi^®^ transformed with plasmid pBM003 was cultured in LB containing 25 μg/mL CM and 0.02% glucose overnight at 37 °C. Overnight cultures were then transferred to Terrific Broth (TB) with 25 μg/mL CM and shaken at 37 °C. When the culture reached an optical density between 0.6 and 0.9, protein expression was induced with 0.2% l-arabinose. Induced cultures were shaken overnight at 37 °C. After centrifugation, the harvested cells were resuspended and protein was extracted from the periplasmic space by the addition of 1 mg/mL polymyxin B. The cell extract was collected by centrifugation and phenylmethylsulfonyl fluoride (PMSF) was added at 100 μM. The extract was purified using cobalt affinity column chromatography (HisPur™ Cobalt Resin, Thermo Fisher Scientific, Waltham, MA, USA) per the manufacturer’s instructions. Fractions were collected in 1.5 mL aliquots and confirmed using SDS-PAGE. Fractions containing ArtABHIS were pooled and dialyzed using 12,000 Da-molecular-weight cutoff (MWCO) dialysis cassettes (Slide-A-Lyzer™, Thermo Fisher Scientific) against 1× PBS + 5% glycerol at 4 °C overnight with one buffer change after 6 h. Dialyzed sample was further concentrated using a 50,000 Da MWCO concentration filter (Amicon Ultra-15, Merck Millipore Ltd., Thermo Fisher Scientific). Molecular weight and purity were confirmed using SDS-PAGE and Western blot with α-HIS_6_ primary (1:2500 Abcam, Cambridge, MA, USA) and HRP-conjugated goat α-rabbit IgG secondary (1:5000, Promega, Madison, WI, USA) antibodies. Protein concentration was determined by bicinchoninic acid (BCA) protein assay (Pierce™ BCA Protein Assay Kit, Thermo Fisher Scientific) using bovine serum albumin (BSA) as a standard.

For purification of native ArtAB and native ArtB, *E. coli* ClearColi^®^ was transformed with plasmid pBM006 or pLC001and induced protein isolated from the *E. coli* periplasmic space as above for pBM003. The extract was purified using immobilized d-galactose affinity column chromatography (Pierce™ d-galactose Agarose, Thermo Fisher Scientific), as described [67]. Column equilibration and washing were performed using 1× PBS, and elution was performed using 1 M d-galactose (ACROS Organics™). Fractions were collected in 1.5 mL aliquots and confirmed with SDS-PAGE. Fractions containing ArtAB or ArtB were pooled and dialyzed in 12,000 Da MWCO dialysis cassettes (Slide-A-Lyzer™, Thermo Fisher Scientific) against 1× PBS + 5% glycerol at 4 °C overnight with one buffer change after 6 h. Dialyzed samples were concentrated using a 50,000 Da MWCO disposable filter (Amicon Ultra-15, Merck Millipore Ltd., Thermo Fisher Scientific). Molecular weight and purity were confirmed with SDS-PAGE, and protein concentration was determined by BCA protein assay (Pierce ™ BCA Protein Assay Kit, Thermo Fisher Scientific) using BSA as a standard. Protein sequences were confirmed by LC–MS (Supplementary Figure S2, Table S3).

### 5.5. ELISA Binding Assays

Flat-bottomed 96-well plates (Nunc MaxiSorp™, Invitrogen, Thermo Fisher Scientific) were coated with either 10 μg/mL fetuin in 1× PBS or 0.15 μM GM1 in DMSO + 1× PBS. Plates were incubated overnight at room temperature. After washing and blocking plates for 1 h in blocking buffer (1% skim milk powder + 0.05% Tween-20), the toxins CT (List Biological Labs, Inc., Campbell, CA, USA) or ArtABHIS were added to the first well at a concentration of 20 μg/mL or 10 μg/mL. Serial two-fold dilutions into blocking buffer were performed and plates were incubated for 2 h at 37 °C. After washing, rabbit anti-6XHIS antibodies (Bethyl Laboratories, Inc., Montgomery, TX, USA) or rabbit anti-CT antibodies (Sigma-Aldrich, St. Louis, MO, USA) were added at 1:5000 dilution and incubated at 37 °C for 1 h. After washing, goat anti-rabbit HRP-conjugated antibodies (Pierce^®^, Thermo Fisher Scientific) were added and incubated at 37 °C for 1 h. Plates were developed with tetramethylbenzidine (TMB) (Promega^TM^ TMB One, Thermo Fisher Scientific) and read at 370 nm per TMB manufacturer’s instructions using a BioTek Cytation 3 imager. ArtAB ELISAs were compared to the average of a no-toxin negative control coated with fetuin or GM1 and using anti-6XHIS antibody. Titers were defined as the reciprocal of the highest dilution at a 370 nm absorbance cut off of 0.2. Titers represent the averages of four independent assays performed in triplicate for ArtAB, or the average of four independent assays for CT.

### 5.6. Cell Culture

African green monkey kidney (Vero; ATCC, Manassas, VA, USA) cells were cultured in Dulbecco’s modified Eagle’s medium (DMEM) (Corning^®,^ Thermo Fisher Scientific) supplemented with 10% fetal bovine serum (FBS; HyClone™, Thermo Fisher Scientific), or bovine growth serum (BGS; HyClone™, Thermo Fisher Scientific) and 1% penicillin/streptomycin (Thermo Fisher Scientific). CHO-K1 cells (CHO; ATCC^®^) were cultured in Ham’s F-12 medium (Corning^®^, Thermo Fisher Scientific) supplemented with 10% FBS and 1% penicillin/streptomycin. Cells were maintained in T-flasks at 37 °C in a humidified 5% CO_2_ incubator and were passaged at 80% confluency using 0.05% trypsin/EDTA. Serum-free cell starvation procedures were completed with media (DMEM for Vero cells, Ham’s F-12 for CHO-K1 cells) supplemented with 1% penicillin/streptomycin but without FBS/BGS.

### 5.7. Fluorescence Microscopy

Internalization assays were performed as described [67]. Briefly, Vero cells were grown 48 h to subconfluence on uncoated coverslips at 37 °C with 5% CO_2_. Cells were maintained in DMEM containing 4 mM L-glutamine, 4500 mg/L glucose, 10% BGS, 100 IU/mL penicillin, and 100 μg/mL streptomycin (DMEM+10). Cells were washed with 1× PBS, then incubated for 15 min at 4 °C, prior to the addition of 50 μg/mL purified ArtABHIS (from *E. coli* ClearColi^®^ + pBM003) or PBS negative control. One half of the cells, including the PBS control, were then shifted to 37 °C for 45 min while the other half remained at 4 °C. Cells were washed, fixed, permeabilized, and blocked for 15 h in 10% BGS at 4 °C. Cells were then incubated withmouse monoclonal anti-6XHIS (α-HIS, HIS.H8; 1:5000 Abcam, Cambridge, MA, USA) and FITC-conjugated anti-mouse IgG (Sigma-Aldrich) antibodies. Coverslips were washed and mounted with hard-set mounting medium with DAPI (Vector Laboratories, Burlingame, CA, USA) before visualization using a Zeiss LSM 510 META laser scanning confocal microscope. Images were acquired using a 100× Alpha Plan-Fluar 1.45 oil DIC objective, a factory set diode laser for DAPI (405 nm), and an argon laser for FITC (488 nm). Image acquisition and processing was performed using the LSM 510 META software. All confocal and image settings were held constant between samples for comparison. Images are representative of at least two fields from one independent assay.

### 5.8. Cellular Activity and Morphology Assays

Cellular activity was assessed using alamarBlue™ and crystal violet assays. 96-well plates were seeded with Vero or CHO-K1 cells at a density of 5000 cells per well in 200 μL of complete culture media and incubated overnight. Cells were starved in serum-free media for 4 h prior to treatment. ArtAB, or ArtB, in a buffer of 1× PBS + 5% glycerol at a concentration of 10 μg/mL (2 μg total in 200 μL), 25 μg/mL (5 μg total in 200 μL), or 50 μg/mL (10 μg total in 200 μL); or CT (List Biological Labs, Inc) in a buffer of 1× PBS at a concentration of 10 μg/mL (2 μg total in 200 μL); or PT (List Biological Labs, Inc.) in a buffer of 1× PBS at a concentration of 10 μg/mL (2 μg total in 200 μL); or respective buffer alone (vehicle) in complete culture media was added to the cells. Each treatment was added to 6 wells per plate. One replicate included an individual plate for each of four post-dose time points. Plates for one replicate were treated at the same time, incubated, and removed from the incubator at the appropriate time point (4, 24, 48, or 72 h post-treatment) for analysis. Three replicates each were completed on Vero cells and CHO-K1 cells. Statistical analysis was completed on one representative replicate (plate) for each cell line (*n* = 6).

After 4, 24, 48, or 72 h of incubation with treatment, brightfield microscopy images at 20× magnification were collected using a BioTek Cytation 3 imager. Images were collected randomly from varied locations within wells and from multiple wells for each treatment dose. Immediately after imaging, 200 μL of 10% (*v/v*) alamarBlue™ (Invitrogen™) in complete culture media was added to each well and plates were incubated for 4 h. Fluorescence was read on the BioTek Cytation 3 imager using excitation wavelength of 560 nm and emission wavelength of 590 nm per AlamarBlue™ manufacturer’s instructions. Light microscopy image files were analyzed using ImageJ Download for Mac OS X. Available online: https://imagej.nih.gov/ij/download.html (Accessed on 22 July 2021). For each image, scale was set according to the pixel:length relationship of the scale bar. Cell length was measured on adherent cells only with a line drawn between the two most distant edges and centered through the middle of the cell body. Dendrites were followed as long as the line could be kept centered in the cell body. Measurements were collected for ten cells in each of three or four frames where each frame represented an independent well for a total of 30 measurements per treatment group. 

After collection of fluorescence data, alamarBlue™ and media were removed and cells were rinsed 3 times with 200 μL 1× PBS. Crystal violet assays were performed per Cold Spring Harbor Protocols [40]. Briefly, 50 μL of crystal violet solution was added to each well and the plates were incubated for 20 min at room temperature with agitation on an orbital shaker. Crystal violet solution was removed and the cells were rinsed 3 times with deionized water with care given not to lift cells from the plates. Plates were air dried overnight. The next day, 100 μL methanol was added to each well and the plate was incubated for 20 min at room temperature with agitation on an orbital shaker. Absorbance at 570 nm was read on the BioTek Cytation 3 imager.

## Data Availability

The data presented in this study are available in the article or supplementary material and are available on request from the corresponding author.

## Supplementary Materials

The following are available online at https://www.mdpi.com/article/10.3390/toxins13090599/s1, Figure S1: Full-length and truncated artAB on different Salmonella serovars. Figure S2: Purification of ArtABHIS from E. coli, and sequencing of ArtB-HIS and native ArtA; Figure S3: Cellular effects of PT on CHO cells; Figure S4: Cell length measurements; Table S1: Protein accession numbers for phylogenetic analysis; Table S2: LC–MS protein identification.
