# Supplementary material for: Cellular Activity of Salmonella Typhimurium ArtAB Toxin and Its Receptor-Binding Subunit
Source: Toxins (Basel). 2021 Aug 27;13(9):599. doi: 10.3390/toxins13090599 (PMC8472264; doi:10.3390/toxins13090599)
Supplement: Supplementary file 1 [file toxins-13-00599-s001.zip › toxins-1294684-supplementary.pdf]

Supplementary Materials: Cellular Activity of Salmonella Typhimurium ArtAB Toxin and Its Receptor-Binding Subunit

Elise Overgaard, Brad Morris, Omid Mohammad Mousa, Emily Price, Adriana Rodriguez, Leyla Cufurovic, Richard S. Beard and Juliette K. Tinker

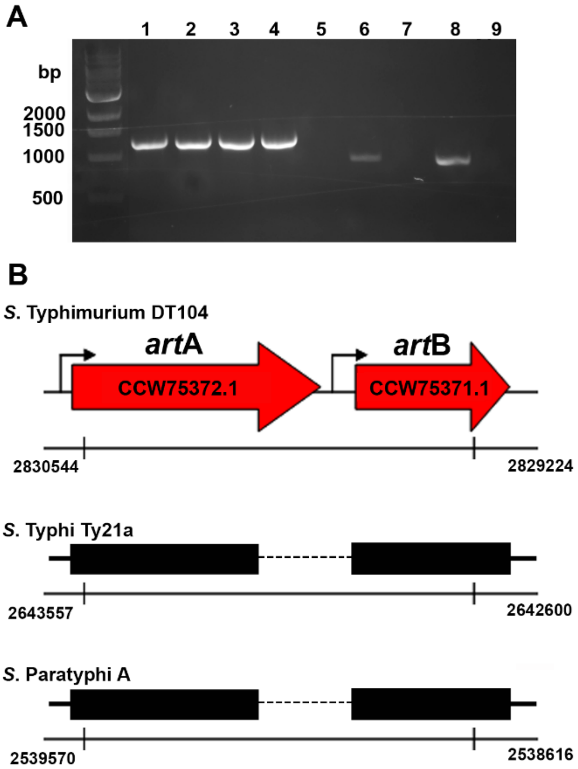

Figure S1. Full length and truncated *art*AB on different *Salmonella* serovars. (**A**) PCR of *S. enterica* serovars Typhimurium DT104 (lanes 1–4; 1320 bps), and Typhimurium non-DT104 (lane 5; no amplification), Typhi (lane 6; 957 bps), Choleraesuis (lane 7; no amplification), and Paratyphi A (lane 8; 954 bps). Lane 9, negative control. (**B**) Diagram of the *art*A – *art*B region in *S.* Typhimurium DT104 (NCBI HF937208), *S.* Typhi Ty21a (NCBI CP023975), and S. Paratyphi A (NCBI CP019185). Serovars Paratyphi, Typhi and Montevideo are positive for *art*B and a truncated version of *art*A (pseudogene) respectively.

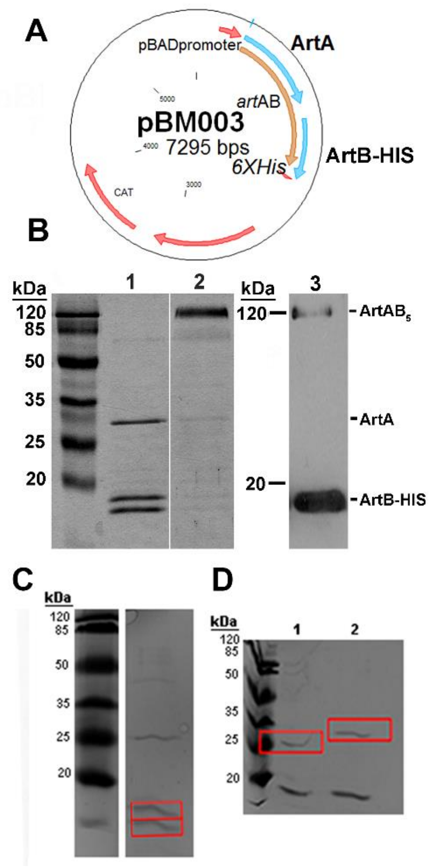

Figure S2. Purification of ArtABHIS from *E. coli*, and sequencing of ArtB-HIS and native ArtA. (**A**) plasmid pBM003 for the expression of ArtAB-HIS; (**B**) SDS-PAGE of: *1*) purified ArtABHIS boiled and *2)* purified ArtABHIS unboiled, and *3)* anti-6XHIS western blot of boiled ArtABHIS (ArtA ≅ 25.6 kDa, ArtB-HIS ≅ 14.2 kDa, $ArtAB_5$-HIS ≅ 96.5 kDa). Bands collected for protein sequencing by mass spectrometry (LC-MS, Supplementary Table S2) included (**C**) ArtB-HIS (from pBM003; top band = pBM003 1, bottom band = pBM003 2) and (**D**) native ArtA (from pBM006; right band = pBM006 1, left band = pBM006 2).

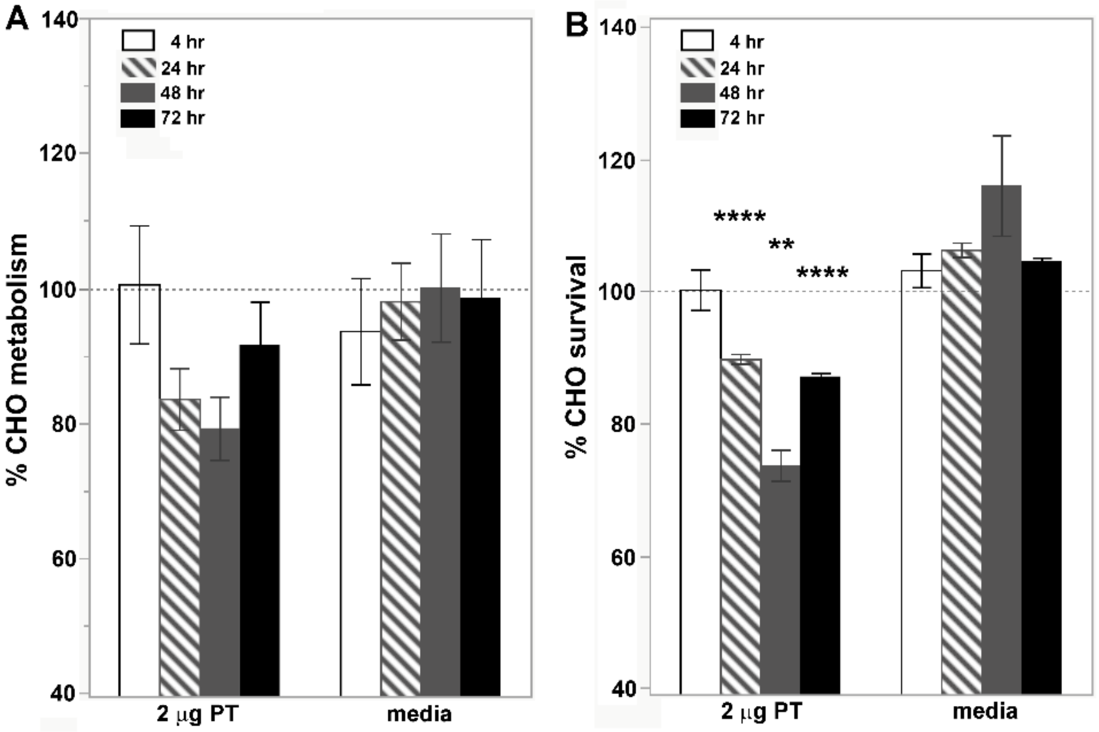

Figure S3. Cellular effects of PT on CHO cells. AlamarBlue™ metabolic assays (**A**) and crystal violet cytotoxic assays (**B**). PT was incubated with cells at 2 μg per 200 μL well for 4, 24, 48, or 72 hours at 37 °C. Results are reported as the percent of vehicle control and the toxin group is compared to media alone at each time point using a one-way analysis of variance (ANOVA) and Tukey-HSD. Stars above the bar indicate a significant difference from media alone at that time point (*$p \leq 0.05$, **$p \leq 0.01$, ***$p \leq 0.001$, ****$p \leq 0.0001$).

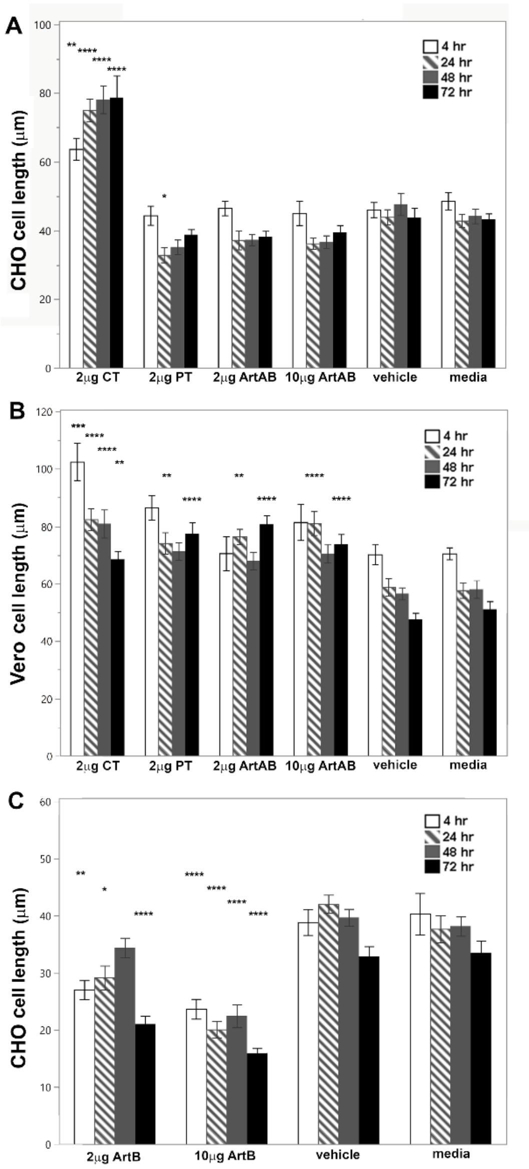

Figure S4. Cell length measurements. (**A**) cell length for CHO cells treated with ArtAB, CT and PT holotoxin, (**B**) cell length for Vero cells treated with ArtAB, CT and PT holotoxin and (C) cell length for CHO cells treated with ArtB subunit. Results are reported as average cell length in μm, and toxin groups and vehicle-treated groups are compared to media alone at each time point using a one-way analysis of variance (ANOVA) and Tukey-HSD. Stars above the bar indicate a significant difference from media alone at that time point (*$p \leq 0.05$, **$p \leq 0.01$, ***$p \leq 0.001$, ****$p \leq 0.0001$).

Table S1. Protein accession numbers for phylogenetic analysis.

Toxin A subunits	NCBI Accession #	Toxin B subunits	NCBI Accession#
ArtA *S.* Typhimurium DT104	WP_156015302	ArtB *S.* Typhimurium DT104	5WHU_A
ArtA *S.* Worthington	BAX76679.1	ArtB *S.* Worthington	BAX76680
ArtA *S. bongori*	ASG55211.1	ArtB *S.* Typhi	AE014613.1
ArtA *S.* Typhi/Montevideo pseudogene	AAMFER010000002	SubB2 *E. coli*	WP_169004307
PltA *S.* Typhi	AE014613.1	ArtB *S. bongori*	BAX76684
PltA/ArtA *E. coli*	WP_001355271	SalB *S. arizonae*	ABX22253.1
PtxA S1 *B. pertussis*	WP_019248344	PltB *S.* Typhi	AE014613.1
YtxA *Y. enterocolitica*	WP_023161032	PtxB S2 *B. pertussis*	WP_050830703
LtIIa *E. coli*	TFY48810	YrpB *Y. pestis*	WP_002209112
LTIIb *E. coli*	1TII_A	SubB *E. coli*	Q6EZC3
EltA (LTA1) *E. coli*	WP_001398470	CfxB *C. freundii*	BAC16522
CtxA *V. cholerae*	1001196A	EcxB *E.coli*	EEU4199622
SubA *E. coli*	WP_000912970	CtxB *V. cholerae*	WP_000593522
CfxA *C. freundii*	BAC16521.1	EltB *E. coli*	WP_024167713
EcxA *E. coli*	WP_071999469	LTIIaB *E. coli*	KDA69244
CdtB *S.* Typhi	NC_003198	LTIIbB *E. coli*	WP_096985491
YrpA *Y. pestis*	WP_064516147	YtxB *Y. enterocolitica*	WP_050137877
ArtA *E. coli*	WP_077887430	Stx2B *E. coli*	EEV2598235
Stx1A *E. coli*	WP_000691354	Stx1B *E. coli*	WP_097586066
Stx2A *E. coli*	EEV2155635.1		

Table S2. LC-MS sequencing of purified proteins.

Protein	Accession	Description	Coverage [%]	# Peptides	# PSMs	# Unique Peptides	# Protein Groups	# AAs	MW [kDa]	calc. pI	Score Sequest HT: Sequest HT	# Peptides (by Search Engine): Sequest HT
pBM003 1	Q404H3	ArtB protein OS=*Salmonella enterica* subsp. enterica serovar Typhimurium str. DT104	48	12	318	12	1	141	15.8	8.05	1047.44	12
pBM003 2	Q404H3	ArtB protein OS=*Salmonella enterica* subsp. enterica serovar Typhimurium str. DT104	48	10	236	10	1	141	15.8	8.05	812.33	10
pBM006 1	Q404H4	ArtA protein OS=*Salmonella enterica* subsp. enterica serovar Typhimurium str. DT104	59	13	317	13	1	241	27.6	8.7	1488.67	13
pBM006 2	Q404H4	ArtA protein OS=*Salmonella enterica* subsp. enterica serovar Typhimurium str. DT104	58	13	496	13	1	241	27.6	8.7	2323.94	13
pLC001	Q404H3	ArtB protein OS=*Salmonella enterica* subsp. enterica serovar Typhimurium str. DT104 OX=85569 GN=ArtB PE=1 SV=1	40	7	60	7	1	141	15.8	8.05	184.77	7
